# Secondary metabolites of *Bacillus subtilis* impact the assembly of soil-derived semisynthetic bacterial communities

**DOI:** 10.3762/bjoc.16.248

**Published:** 2020-12-04

**Authors:** Heiko T Kiesewalter, Carlos N Lozano-Andrade, Mikael L Strube, Ákos T Kovács

**Affiliations:** 1 Bacterial Interactions and Evolution Group, DTU Bioengineering, Technical University of Denmark, Kgs. Lyngby, Denmark; 2 Bacterial Ecophysiology and Biotechnology Group, DTU Bioengineering, Technical University of Denmark, Kgs. Lyngby, Denmark

**Keywords:** *Bacillus subtilis*, bacterial community, chemical ecology, *Lysinibacillus fusiformis*, nonribosomal peptides, surfactin

## Abstract

Secondary metabolites provide *Bacillus subtilis* with increased competitiveness towards other microorganisms. In particular, nonribosomal peptides (NRPs) have an enormous antimicrobial potential by causing cell lysis, perforation of fungal membranes, enzyme inhibition, or disruption of bacterial protein synthesis. This knowledge was primarily acquired in vitro when *B. subtilis* was competing with other microbial monocultures. However, our understanding of the true ecological role of these small molecules is limited. In this study, we have established soil-derived semisynthetic mock communities containing 13 main genera and supplemented them with *B. subtilis* P5_B1 WT, the NRP-deficient strain *sfp*, or single-NRP mutants incapable of producing surfactin, plipastatin, or bacillaene. Through 16S amplicon sequencing, it was revealed that the invasion of NRP-producing *B. subtilis* strains had no major impact on the bacterial communities. Still, the abundance of the two genera *Lysinibacillus* and *Viridibacillus* was reduced. Interestingly, this effect was diminished in communities supplemented with the NRP-deficient strain. Growth profiling of *Lysinibacillus fusiformis* M5 exposed to either spent media of the *B. subtilis* strains or pure surfactin indicated the sensitivity of this strain towards the biosurfactant surfactin. Our study provides a more in-depth insight into the influence of *B. subtilis* NRPs on semisynthetic bacterial communities and helps to understand their ecological role.

## Introduction

In nature, bacteria live in complex communities where they interact with various other microorganisms. Most microbial communities are influencing biochemical cycles and impact agriculture, from which the latter is primarily mediated due to plant-growth promotion [1–4]. Extensive research has been conducted in the last decade to scrutinise the occurring natural processes and their impact on the environment, to investigate the functions and interactions of community members, such as metabolite cross-feeding interactions, and to eventually engineer them [5–7]. The soil is one of the five main habitats of bacteria and archaea [8]. Soil is very heterogeneous since it exhibits spatial variability in terms of nutrient availability and geochemical features [9]. Therefore, soil consists of microbial hotspots, indicating faster process rates than the average soil [10]. One such microbial hotspot is the rhizosphere, harbouring microbial communities where various interactions between bacteria, fungi, and plants take place [11]. The composition of microbial communities depends on multiple factors. Studies have revealed that the composition of bacterial soil communities varies at the same sampling site during different seasons [12–13]. Moreover, it has been recently demonstrated that precipitation rates have a significant impact on bacterial communities since bacterial soil communities have a higher diversity in dry than in rainy seasons [14]. Besides the seasonal factors, even different plant species with varying root exudates as well as various soil types impact the microbial community composition in the rhizosphere [15–20]. Microbial communities can consist of hundreds and thousands of diverse species, which makes investigations very challenging and hard to reproduce. One alternative approach is to establish a host-associated synthetic community, usually with members of the same kingdom, with a defined composition but fewer members [19,21]. Lebeis et al. used an artificial community of 38 bacterial strains to demonstrate that plant phytohormones sculpt the root microbiome [19]. In comparison, Niu et al. established a seven-species bacterial community based on host selection to mimic the principle root microbiome of maize [22].

Secondary metabolites (SMs) are believed to be important mediators of the interactions between microorganisms [23]. Many of them are well-studied in vitro, but the true ecological role of SMs is still the subject of investigations. Different opinions about their primary role in nature exist in the literature; some share the view that SMs are mainly microbial weapons but others instead designate them as signalling molecules [24–27]. Additionally, Pettit [28] and Wakefield et al. [29] have demonstrated in 2009 and 2017, respectively, that some bacterial or fungal biosynthetic gene clusters are silent when strains are grown in monocultures under standard laboratory conditions but are expressed in intra- or interkingdom co- or multicultures. Furthermore, they could show that some SMs had a higher production rate in multicultures, highlighting that neighbouring organisms induce and increase the SM production in the tested strains.

**Figure 1:**
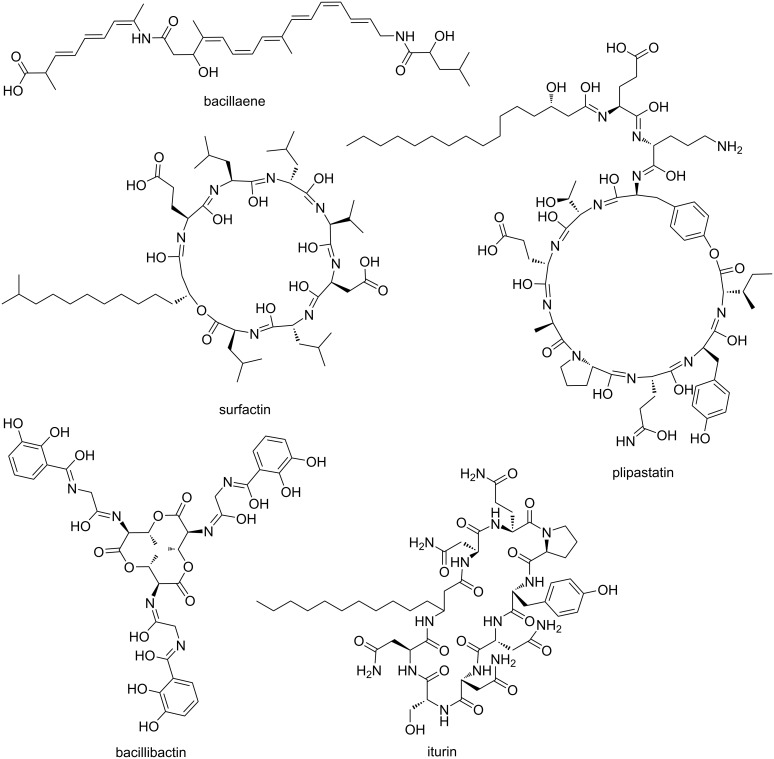
Overview of the NRPs surfactin, plipastatin, bacillibactin, and iturin as well as the hybrid NRP-PK bacillaene produced by *Bacilli*.

*Bacillus subtilis* is a well-studied soil bacterium and is used as a model organism for biofilm formation and sporulation [30]. It has been shown that several members of the *B. subtilis* species complex have exceptional plant growth promoting and plant health improving properties by suppressing plant pathogenic bacteria and fungi [31]. However, it is not completely understood how soil-administered *Bacillus* spp. affect the indigenous microbial communities. Gadhave et al. have shown that the supplementation of *B. subtilis*, *Bacillus amyloliquefaciens* (now identified as *Bacillus velezensis*), and *Bacillus cereus* to the roots of broccoli plants led to species-dependent changes in the diversity, evenness, and relative abundances of endophytic bacterial communities [32]. Like many other soil bacteria, *B. subtilis* and other *Bacillus* spp. produce various SMs [33–34]. The most prominent and bioactive SMs are nonribosomal peptides (NRPs), of which isoforms belong to the families of surfactins, fengycins, or iturins [35–36] (Figure 1). They are biosynthesised by large enzyme complexes, nonribosomal peptide synthetases (NRPSs). For the biosynthesis of *B. subtilis* NRPs, the phosphopantetheinyl transferase *Sfp* is needed since it has been shown to activate the peptidyl carrier protein domains, converting it from the inactive apo-form to the active holo-form [37]. *B. subtilis* has four *sfp*-dependent SMs, of which three are synthesised by NRPS gene clusters (surfactin, plipastatin, and bacillibactin) and one by a hybrid NRPS–PKS gene cluster (bacillaene, Figure 1). The well-studied biosurfactant surfactin, encoded by the *srfAA-AD* gene cluster, reduces the surface tension needed for swarming and sliding motility [38–39]. The surfactin bioactivity is specifically evoked by the surfactant activity triggering cell lysis due to penetration of the bacterial lipid bilayer membranes and the formation of ion-conducting channels [40–42]. The bioactivity of surfactin was shown against *Listeria* spp. and *Legionella monocytogenes* [43–44]. It is presumed that the antifungal plipastatin, expressed from the *ppsA-E* gene cluster, acts as an inhibitor of phospholipase A2, forming pores in the fungal membrane and causing morphological changes in the fungal membrane and cell wall [45–46]. This antifungal potential was demonstrated primarily against various filamentous fungi [47–51]. The broad-spectrum antibiotic bacillaene, synthesised by the *pksB-S* gene cluster, is mainly targeting bacterial protein synthesis [52]. Still, it was also shown that it could protect cells and spores from predation [53]. We recently demonstrated that the production of these NRPs varies among coisolated *B. subtilis* environmental strains due to missing core genes or potentially altered gene regulation, highlighting the existing natural diversity of SM production in this species [51].

In this study, we focus on soil-derived semisynthetic bacterial mock communities and describe how these are affected by a *B. subtilis* strain that was previously isolated from the same sampling site from which the bacterial mock communities originated. With an NRP-mutant-based approach, we investigated the impact of NRPs on the establishment and composition of the bacterial communities. We previously demonstrated that the strain P5_B1 produces the NRPs surfactin and plipastatin and has further BGC predictions for the NRPs bacillaene and bacillibactin [51]. It was revealed by 16S rRNA amplicon sequencing that the established semisynthetic mock communities contained 13 genera with a relative abundance of >0.19% in at least one mock community. Furthermore, it was demonstrated that the addition of *B. subtilis* suppressed the genera *Lysinibacillus* and *Viridibacillus*. Additional optical density (OD)-based growth monitoring of the selected strain *Lysinibacillus fusiformis* M5 confirmed the impact of *B. subtilis*-produced surfactin on the growth.

## Results

### Impact of *B. subtilis* secondary metabolites on taxonomic groups in semisynthetic mock communities

**Figure 2:**
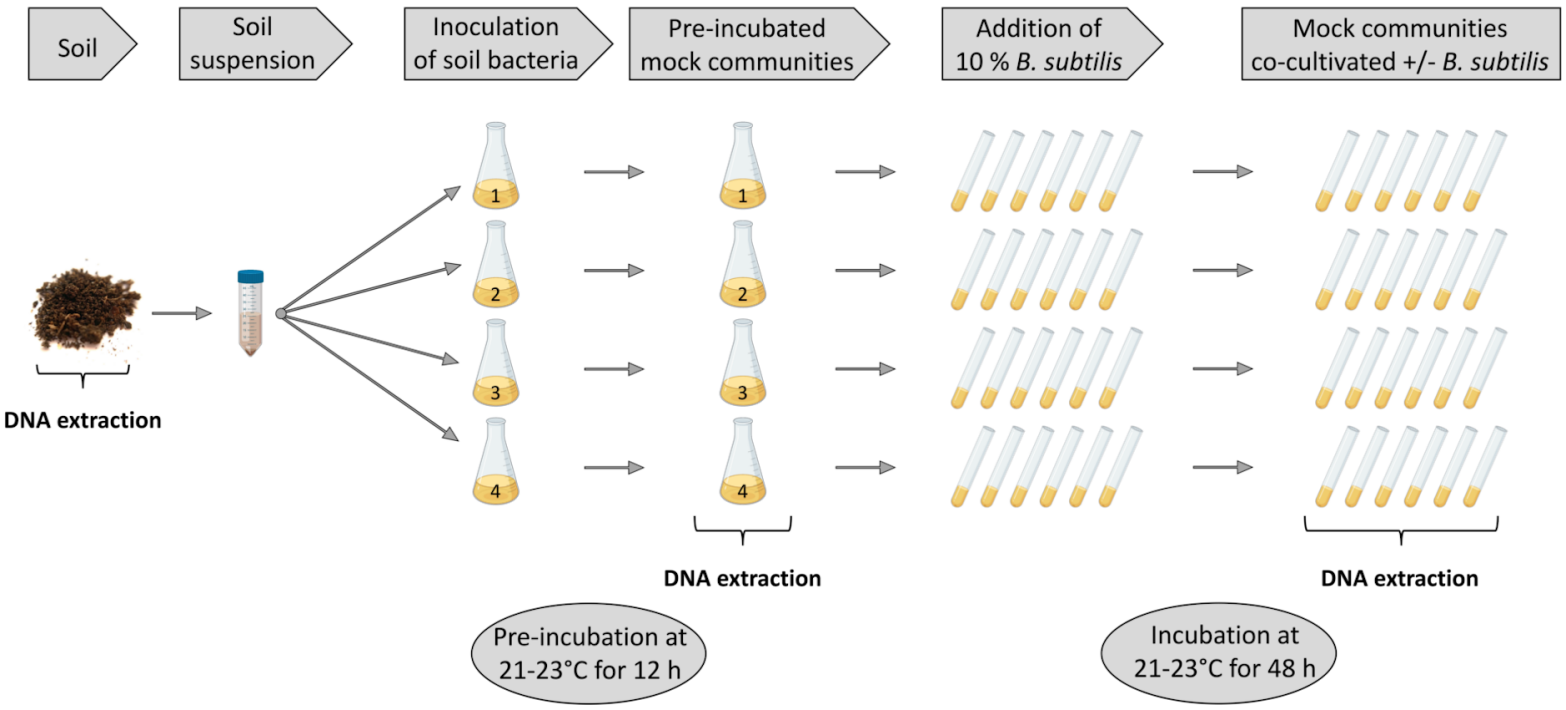
Overview of the experimental setup. A soil suspension, obtained from a soil sample, was used as an inoculum for four independent replicates and preincubated for 12 h. Enriched precultures were aliquoted and supplemented with 10% *B. subtilis* strains or left untreated and incubated for 48 h. DNA was extracted from the soil sample, preincubated soil suspensions, and mock communities. Parts of this figure were created using BioRender.com.

**Figure 3:**
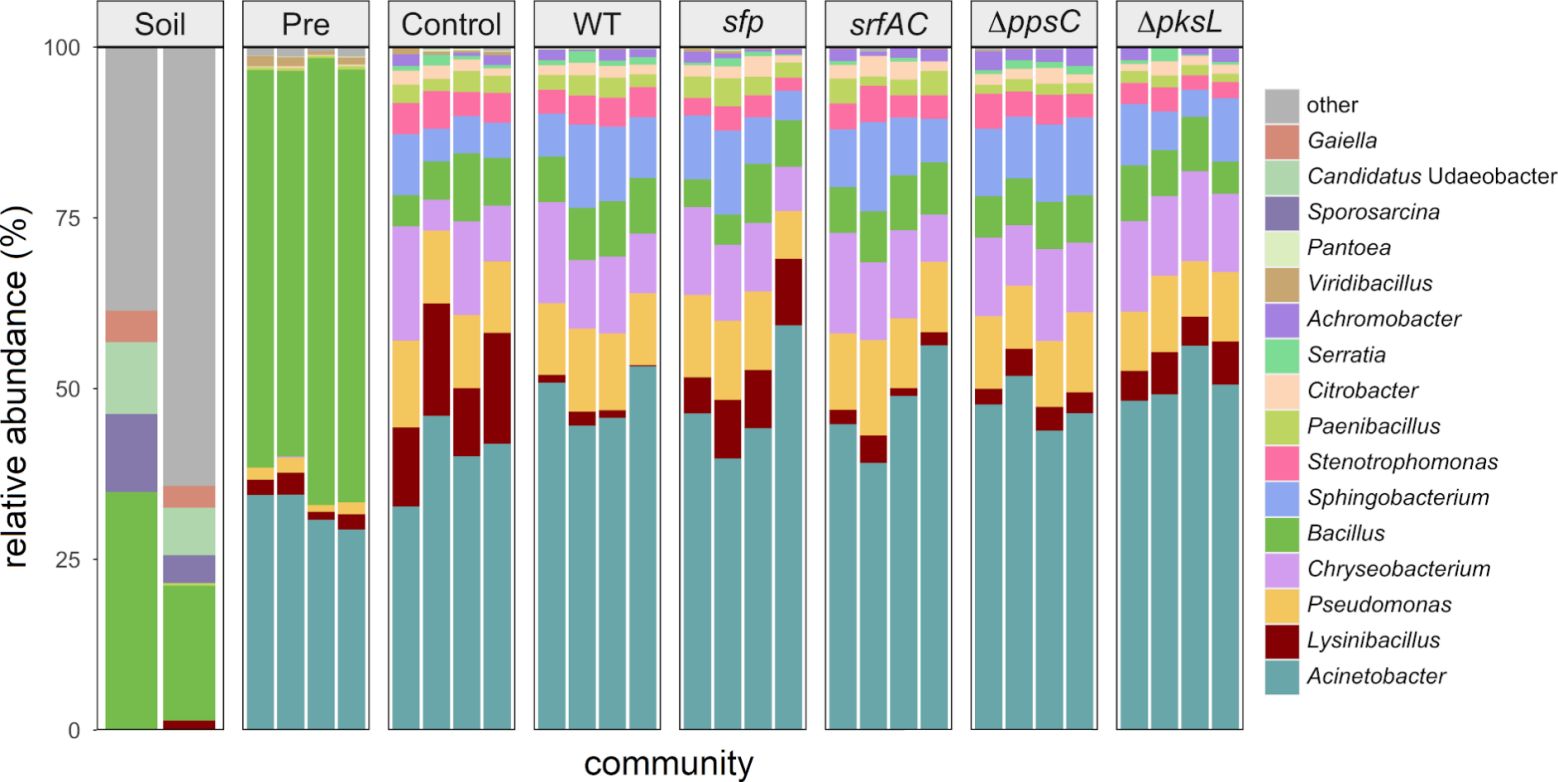
The taxonomic summaries are showing the relative abundance of the most abundant genera for each replicate of the soil sample (“soil”), 12 h preincubated soil suspensions (“Pre”), and untreated (“Control”) or treated mock communities with either *B. subtilis* wild type (“WT”), the NRP-deficient strain *sfp*, the surfactin mutant *srfAC*, the plipastatin mutant ∆*ppsC*, or the bacillaene mutant ∆*pksL*, cocultivated for 48 h. Genera are classified as “other” when the relative abundance is <2% (“Soil”), <1% (“Pre”), or <0.19% (in all differently treated mock communities).

We established soil-derived semisynthetic mock communities and supplemented them with *B. subtilis* WT P5_B1, the corresponding NRP-deficient strain *sfp*, or the single-NRP mutants *srfAC*, ∆*ppsC*, and ∆*pksL*, respectively, incapable of producing either surfactin, plipastatin, or bacillaene, and kept the untreated culture as a control (Figure 2). To investigate the impact of *B. subtilis* NRPs on the bacterial community composition, we sequenced and analysed amplicons of the V3-V4 region of the 16S rRNA gene. The taxonomic summaries give an overview on the relative abundance of the most frequent genera present in each assay and replicate (Figure 3). We investigated the taxonomic level genus since we could not observe any differences among the treated and untreated communities at the class level and similar observations between the family and the genus levels. Moreover, the targeted V3-V4 region of the 16S rRNA gene does not allow sufficient distinction below this rank. Unsurprisingly, the two soil samples differed tremendously from the in vitro samples and indicated a higher genus richness. We determined that *Bacillus* was the most abundant genus in the two soil samples, with a relative proportion between 19% and 35%. Other genera with an abundance higher than 2% were *Sporosarcina* (4–11%), *Candidatus* Udaeobacter (7–10%), and *Gaiella* (3–4%). The communities of the 12 h precultivated soil suspension consisted primarily of the two genera *Bacillus* (56–65%) and *Acinetobacter* (29–34%). Additional genera with an abundance higher than 1% were *Lysinibacillus* (1.2–3.2%), *Pseudomonas* (1.0–2.2%), and *Viridibacillus* (0.6–1.5%). The genus richness of the four precultured soil suspensions was between 12 and 18 of the total 21 genera.

**Figure 4:**
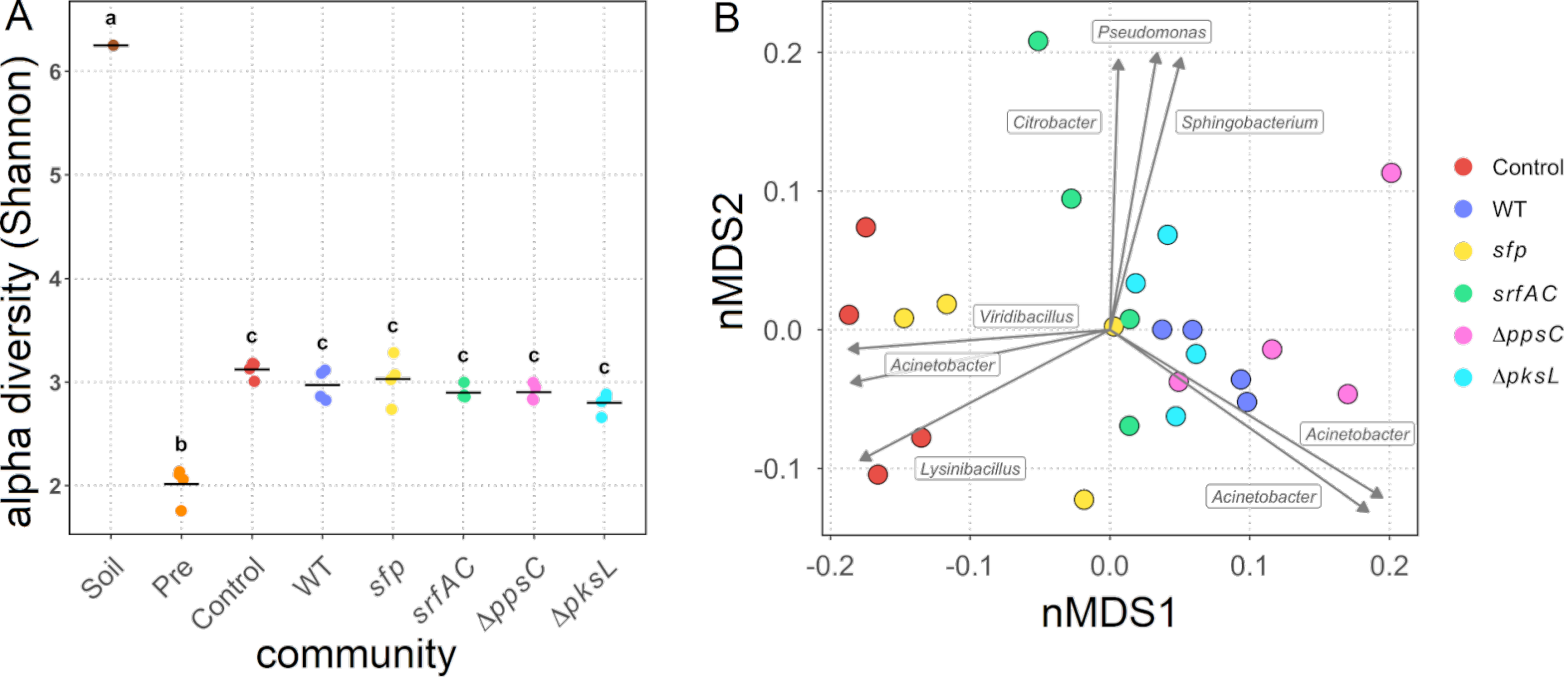
Diversity analyses of the soil sample (“Soil”), 12 h preincubated soil suspensions (“Pre”), and untreated (“Control”) or treated mock communities with either *B. subtilis* wild type (“WT”), the NRP-deficient strain *sfp*, the surfactin mutant *srfAC*, the plipastatin mutant ∆*ppsC*, or the bacillaene mutant ∆*pksL*, cocultivated for 48 h. A) Alpha diversity (in Shannon) of the different samples. Each point represents a replicate, while the line indicates the mean of the Shannon diversity indexes. B) Beta diversity of the mock communities calculated with the Bray–Curtis dissimilarity and visualised as circles in a nMDS. The vectors, each labelled with the corresponding genus, represent the ASVs, with the highest correlating with the nMDS ordination. The vector lengths are proportional to the level of correlation.

Diversity analyses were performed to determine the overall impact of the NRPs on the diversity of the bacterial mock communities. The read numbers varied among the different samples (Table S3, Supporting Information File 1), but we had to exclude the sample “Soil 1” from the analysis since it had the lowest read number, and the rarefaction curve was not reaching a clear asymptote (Figure S1, Supporting Information File 1). The alpha diversity revealed that the mock communities cocultivated for 48 h had Shannon indexes between 2.7 and 3.3, and thus a similar genus richness and evenness (Figure 4A). However, it also highlighted that the precultivated communities had the lowest Shannon indexes between 1.8 and 2.1. Consequently, these communities have a lower species evenness and are therefore dominated by fewer species. The soil sample had the highest Shannon index (6.3), which highlights that the richness and evenness are expectedly larger than in the in vitro communities. The alpha diversity of the soil sample and preincubated soil suspensions differed from the mock communities, but we could not see differences between the mock communities.

Therefore, we determined the beta diversity only for the treated and untreated mock communities cocultivated for 48 h. The analysis underlined a high similarity in the composition of the mock communities treated with *B subtilis* strains (Figure 4B). However, the control mock communities separated from the majority of the treated communities along the nonmetric multidimensional scaling 1 (nMDS1) axis. Interestingly, two replicates of the *sfp*-treated communities had a low Bray–Curtis dissimilarity to the control communities, emphasising a high similarity to the untreated control communities. In contrast, the communities supplemented with NRP-producing *B. subtilis* strains clustered together and indicated a lower dissimilarity to each other than to the control communities. Notably, the communities treated with the *srfAC* mutant had a higher dispersion, likely owing to a low number of reads in two of the replicates. We fitted the most correlating (*R*^2^ > 0.6) amplicon sequence variants (ASVs) to the nMDS ordination and plotted them as vectors to investigate the differences between the mock communities. The analysis indicated that three ASVs, taxonomically assigned to the genera *Lysinibacillu*s, *Acinetobacter*, and *Viridibacillus*, correlated with the control and two *sfp*-treated communities. This observation suggests that the absence of NRP-producing *B. subtilis* resulted in an increased abundance of these. Furthermore, two ASVs of the genus *Acinetobacter* correlated best with the communities supplemented with the NRP-producing *B. subtilis* strains, hinting a higher frequency of these in NRP-treated communities. Additionally, three ASVs, identified as *Pseudomonas*, *Citrobacter*, and *Sphingobacterium*, correlated with two communities treated with the surfactin mutant. A similar but smaller correlation with two bacillaene mutant-treated communities was detectable as well. These results imply a negative impact of either surfactin or bacillaene on the four ASVs. Interestingly, the vector-based analysis suggests that, depending on the ASVs, the genus *Acinetobacter* is both positively and negatively affected by the NRPs.

In conclusion, the alpha diversity analyses revealed that species richness and evenness were reduced in the in vitro communities compared to the soil community. Furthermore, 12 h preincubated soil suspensions showed a reduced diversity compared to the mock communities incubated for 48 h. Nevertheless, we could not detect an effect of the supplemented *B. subtilis* strains on the diversity. However, the beta diversity results suggested that the addition of NRP-producing *B. subtilis* strains influenced the composition of the mock communities. Mainly ASVs belonging to the genera *Lysinibacillus*, *Viridibacillus*, and *Acinetobacter* were affected by the presence or absence of *B. subtilis* NRPs in the bacterial mock communities.

The diversity, in particular the species evenness, increased independently of the treatment in all established mock communities, compared to the precultivated soil suspensions and contained 11–18 genera (Figure 3). The most abundant genera, having a proportion greater than 0.19% in at least one *B. subtilis*-treated or untreated mock community were *Acinetobacter*, *Lysinibacillus*, *Pseudomonas*, *Chryseobacterium*, *Bacillus*, *Sphingobacterium*, *Stenotrophomonas*, *Paenibacillus*, *Citrobacter*, *Serratia*, *Achromobacter*, *Viridibacillus*, and *Pantoea*. Noteworthy, the prevalence of the *Bacillus* genus was comparable in the *B. subtilis*-treated communities (4–9%) and the control (5–10%). In the latter, the present *Bacillus* ssp. originated only from the soil suspension, highlighting that the additional supplementation of *B. subtilis* did not affect the relative abundance of the genus *Bacillus* after 48 h cocultivation. Interestingly, the only genera detected in both the in vitro mock communities and the soil samples were *Bacillus*, *Lysinibacillus*, and *Paenibacillus.* The remaining most abundant genera in the mock communities were below the detection limit.

**Figure 5:**
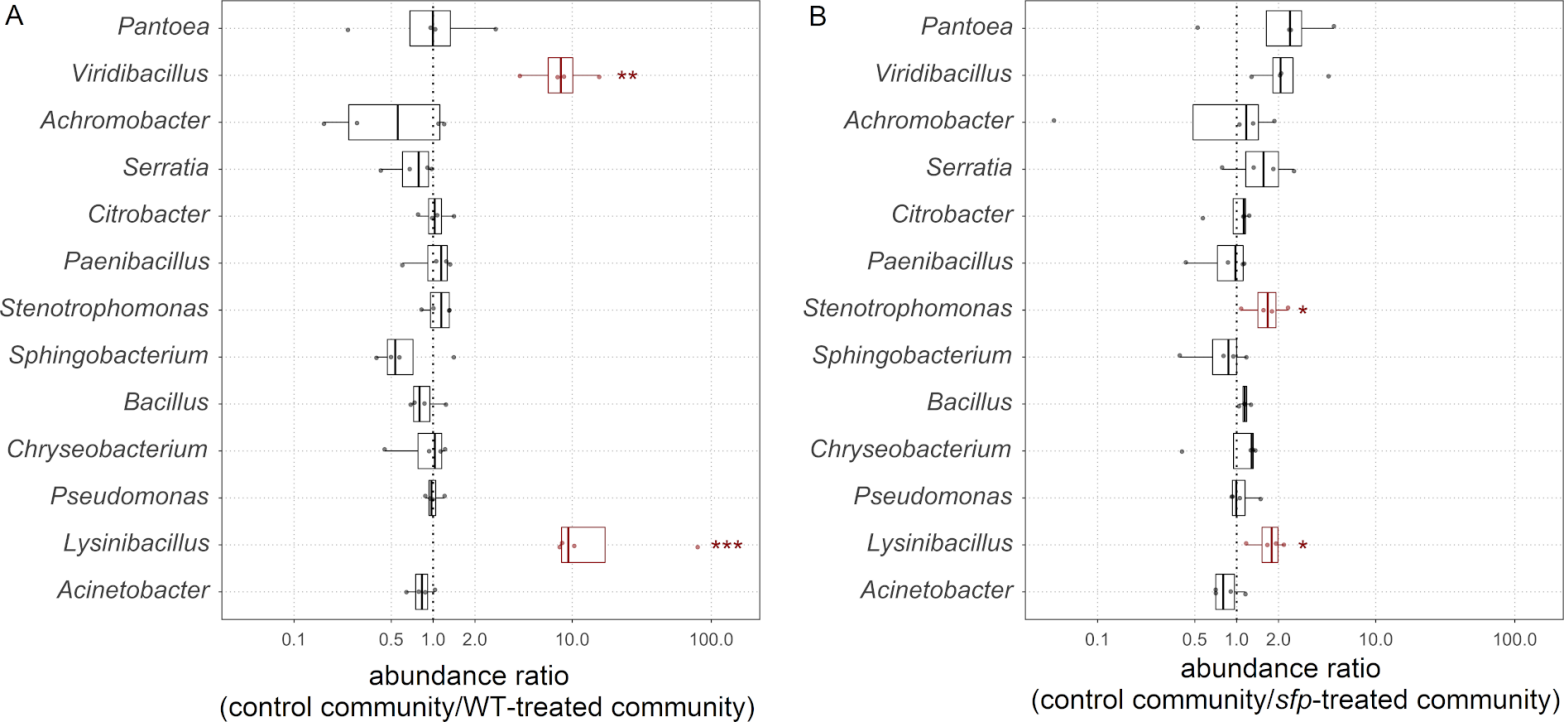
Abundance ratios for each genus and replicate (points) in the control community compared to the WT-treated (A) and to the *sfp*-treated community (B). Red-box plots highlight the statistical significance, which is defined as *P* ≤ 0.05 (*), *P* ≤ 0.01 (**), and *P* ≤ 0.001 (***).

The comparison of the abundance ratios between the control communities and *B. subtilis* WT-treated communities revealed that *Lysinibacillus* and *Viridibacillus* were significantly decreased 9.4-fold (*P* ≤ 0.001) and 8.3-fold (*P* ≤ 0.01), respectively, in the communities supplemented with *B. subtilis* WT (Figure 5A)*.* None of the other genera was affected by the addition of this strain. In comparison, we could only detect a 1.8-fold significant reduction (*P* ≤ 0.05) of *Lysinibacillus* in the *sfp*-treated communities compared to the untreated communities, and thus a greatly diminished effect compared to the WT-treated samples was evident (Figure 5B). Also, we could not observe a significant reduction of *Viridibacillus*, but besides *Lysinibacillus*, also *Stenotrophomonas* was 1.7-fold (*P* ≤ 0.05) significantly reduced in these communities. The direct comparison of WT- and *sfp*-treated communities confirmed the NRP-dependent suppression of both *Lysinibacillus* and *Viridibacillus* in the WT-treated communities and the suppression of *Stenotrophomonas* in the *sfp*-treated communities (Figure S2, Supporting Information File 1).

**Figure 6:**
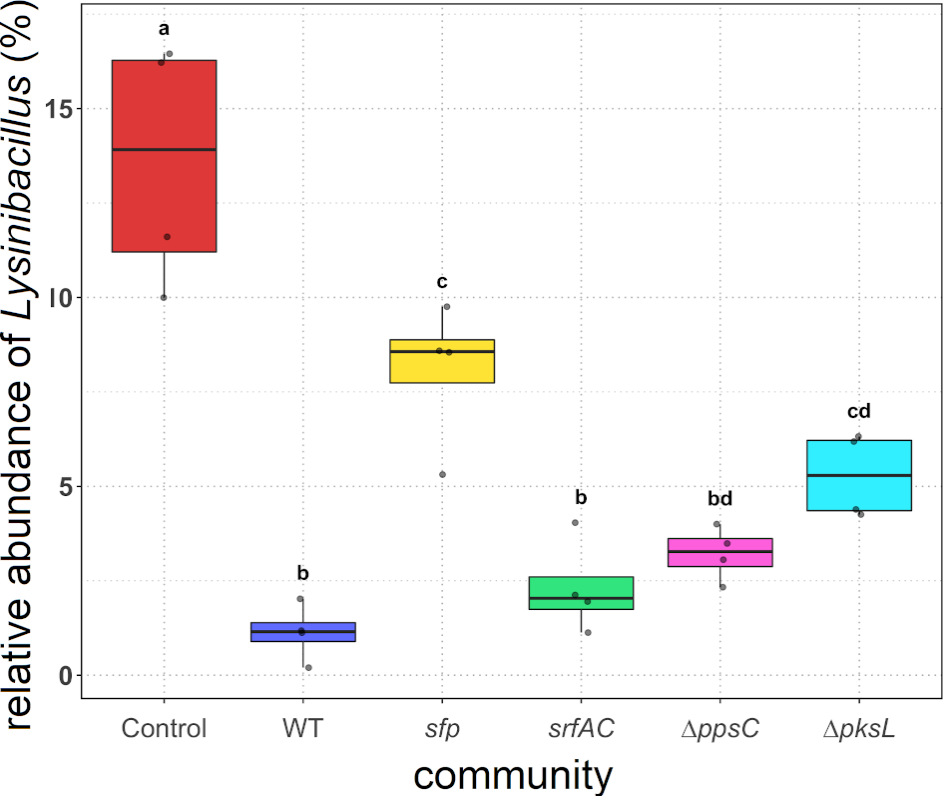
The relative abundance of *Lysinibacillus* in the untreated (“Control”) and treated mock communities with either *B. subtilis* wild type (“WT”), the NRP-deficient strain *sfp*, the surfactin mutant *srfAC*, the plipastatin mutant ∆*ppsC*, or the bacillaene mutant ∆*pksL*, cocultivated for 48 h. The points represent the abundance in each replicate. Treatments with different letters are significantly different (*P* ≤ 0.05).

Concentrating on *Lysinibacillus*, the highest abundance of this genus was discernible in the control assays (13.9%), which was significantly different compared to all other *B. subtilis*-treated assays (Figure 6). However, when *B. subtilis* P5_B1 WT was added to the mock communities, a significant decrease (*P* ≤ 0.001) of *Lysinibacillus* (1.2%) compared to the control communities was discovered. Furthermore, when we added the NRP-deficient strain *sfp*, we could notice a significantly higher abundance of *Lysinibacillus* (8.6%) compared to the WT-treated communities (*P* ≤ 0.001) but still a significantly lower prevalence compared to the control communities (*P* ≤ 0.05). Compared to the WT-treated communities, the frequency of *Lysinibacillus* was slightly but not significantly higher in the communities treated with the single-NRP mutants *srfAC* (2.0%) and ∆*ppsC* (3.3%). The abundance of *Lysinibacillus* in the assays containing the ∆*pksL* strain (5.3%) was significantly higher (*P* ≤ 0.01) than in the WT-treated assays. However, the *Lysinibacillus* abundance in ∆*pksL*-treated communities was not significantly different from the ∆*ppsC*- or *sfp*-treated communities. In summary, *Lysinibacillus* was affected by the addition of *B. subtilis* independent of the NRPs, but when *B. subtilis* strains capable of producing them were present, the impact on *Lysinibacillus* was enhanced. Furthermore, the results indicate that bacillaene had the strongest and surfactin the weakest effect on *Lysinibacillus* in the mock communities.

The second genus affected by the addition of *B. subtilis* was *Viridibacillus*, which had a very low abundance in the control mock communities (0.49%) compared to *Lysinibacillus* (Figure S3, Supporting Information File 1). However, when *B. subtilis* WT was added to the community, *Viridibacillus* indicated a significantly lower (*P* ≤ 0.01) abundance (0.03%) compared to the control communities. Notably, in two of the WT-treated community replicates, *Viridibacillus* was below the detection level. Nevertheless, the abundance of this genus in the *sfp*-treated communities (0.26%) was statistically not significant in comparison to the WT and the control communities. Furthermore, the addition of the single-NRP mutants *srfAC*, ∆*ppsC*, and ∆*pksL* resulted in communities with *Viridibacillus* frequencies similar to the WT-treated communities (0.08%, 0.05%, and 0.00%, respectively). *Viridibacillus* as well as *Lysinibacillus* was affected by the addition of *B. subtilis* to the communities. However, no particular NRP could be assigned to the reduced frequency of *Viridibacillus*.

### Growth properties of *L. fusiformis* M5 supplemented with *B. subtilis* spent media

**Figure 7:**
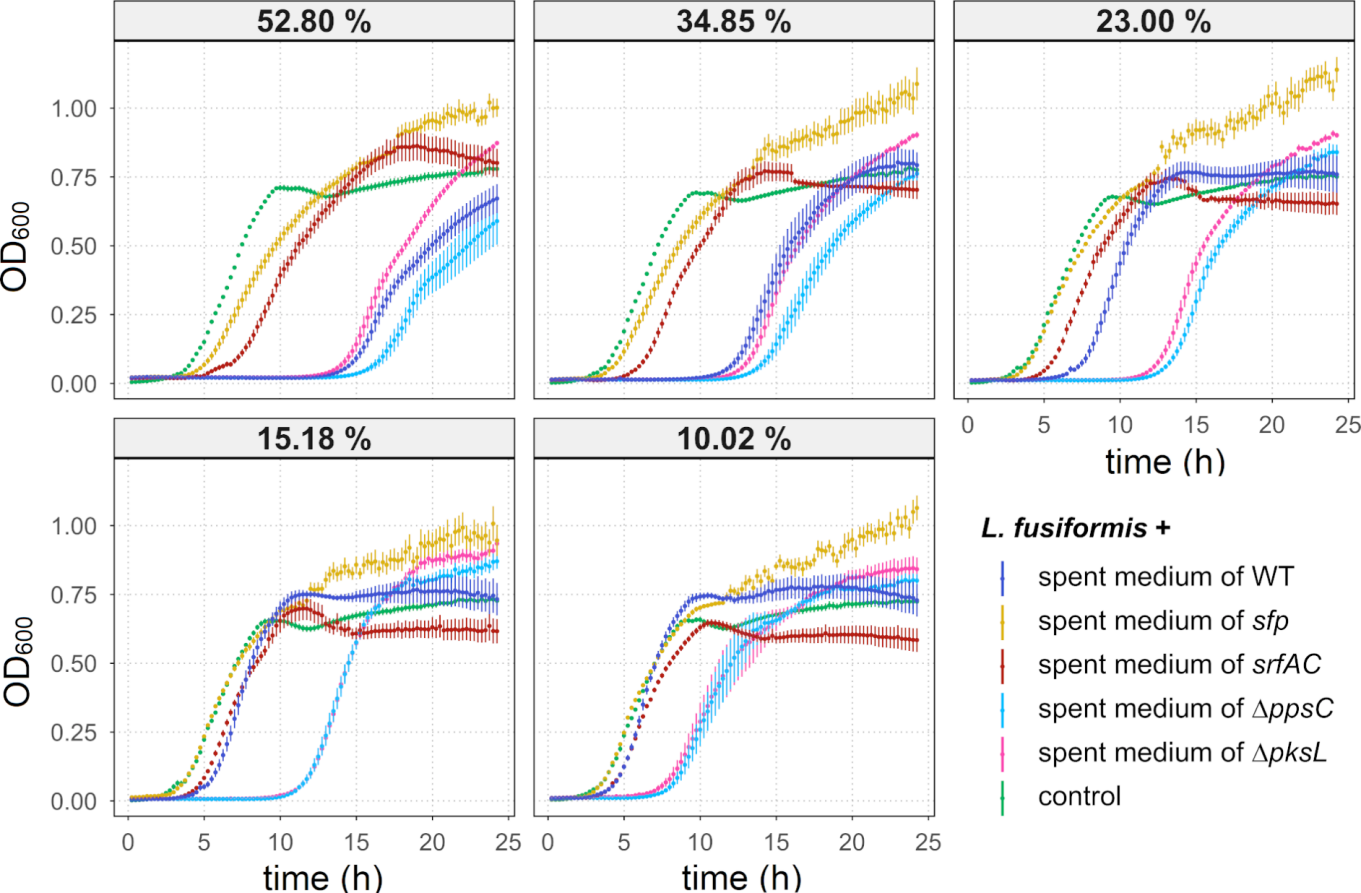
Growth curves of *L. fusiformis* M5 exposed to spent media from 48 h *B. subtilis* cultures and without treatment (“control”). The spent medium concentration of 10.02% to 52.80%, acquired with a serial dilution, indicates the proportion of spent medium from the total volume. The error bars represent the standard error. *N* ≥ 6. OD_600_ = optical density at 600 nm.

The main finding from the semisynthetic mock community experiment indicated that the genus *Lysinibacillus* was negatively affected by the addition of *B. subtilis* P5_B1 WT and that NRPs enhance the suppression. To dissect the direct impact of a particular NRP in this inhibition, we monitored the growth of *L. fusiformis* M5, a previously isolated *Lysinibacillus* species [54], over 24 h when treated with different proportions of spent media from *B. subtilis* WT and the corresponding NRP mutants (Figure 7). When we added 52.80*%* of spent medium to *L. fusiformis,* we observed the fastest entry into the exponential growth phase in the untreated assay. Interestingly, the addition of spent medium of either WT, ∆*ppsC*, or ∆*pksL* caused a delay of entering into this growth phase of approximately 11–13 h in *L. fusiformis* compared to the control. Such a strong effect was not observed when the spent medium of the *sfp* or *srfAC* mutant was added. The addition of these two spent media caused only a slight delay of the exponential growth phase of *L. fusiformis*, although spent *sfp* medium had a lower effect on *L. fusiformis* compared to spent *srfAC* medium. When 23.00% of spent medium was added, no growth differences could be detected anymore between the control and the *sfp*-treated assays in the exponential growth phase. Furthermore, the effect of spent WT medium seems to be reduced at this concentration, but the spent media of ∆*ppsC* and ∆*pksL* maintained their growth inhibition potential. The lowest concentration of a spent medium having an inhibitory effect was 10.02%. At this concentration, only the spent media of ∆*ppsC* and ∆*pksL* affected the growth of *L. fusiformis*, even though it was weakened compared to using higher concentrations. Intriguingly, a higher level of aggregation was observed in the *L. fusiformis* assays supplemented with the spent medium of *sfp* compared to the other assays, which caused higher and variable OD measurements in the stationary phase of the growth curves (Figure S4, Supporting Information File 1). Finally, it was noted that the final cell density was slightly higher in the assays supplemented with the spent medium compared to the control assays.

These results revealed that *B. subtilis*-mediated inhibition of *L. fusiformis* is NRP-dependent since the spent medium of the NRP-deficient strain *sfp* had an only minor impact. Moreover, we hypothesise that surfactin is responsible for the direct inhibitory effect on *L. fusiformis*, as this was the only spent medium of an NRP mutant strain with lowered inhibition compared to spent media of other single NRP mutants.

### Impact of surfactin on the growth of *L. fusiformis*

**Figure 8:**
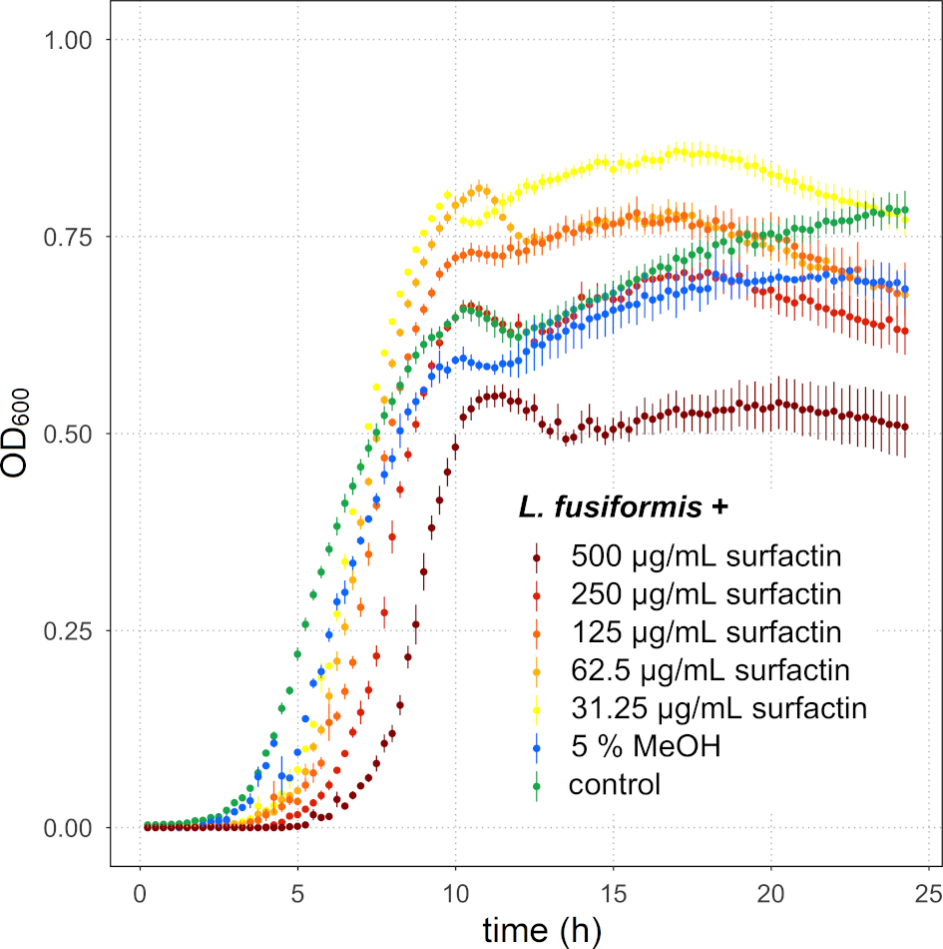
Growth curves of *L. fusiformis* M5 exposed to different concentrations of surfactin, the highest concentration of the solvent MeOH, and without treatment (“control”). The error bars represent the standard error. *N* ≥ 5 (control and surfactin-treated assays), *N* = 2 (MeOH-treated assays).

To confirm the inhibitory effect of surfactin on *L. fusiformis*, we exposed this strain to different concentrations of pure surfactin dissolved in methanol and monitored its growth over 24 h. The growth of *L. fusiformis* was delayed in the exponential growth phase when surfactin was supplemented in concentrations between 31.25 µg/mL and 500 µg/mL (Figure 8). At a surfactin concentration of 500 µg/mL, the cell density in the stationary phase was lower than the control. At a concentration of 250 µg/mL, the cell density reached a level similar to the untreated control. However, when surfactin was added in concentrations between 125 and 31.25 µg/mL, after an initial growth delay into the exponential phase, the cell densities in all treatments exceeded the ones of the control. The highest concentration of the solvent methanol of 5% had only a minor inhibiting effect on *L. fusiformis,* whereas lower concentrations of methanol showed no inhibition (Figure S5, Supporting Information File 1). These results suggest that surfactin has growth inhibitory effects on *L. fusiformis,* and we hypothesise that it might act as the key inhibitory *B. subtilis* NRP under the tested conditions.

## Discussion

**Figure 9:**
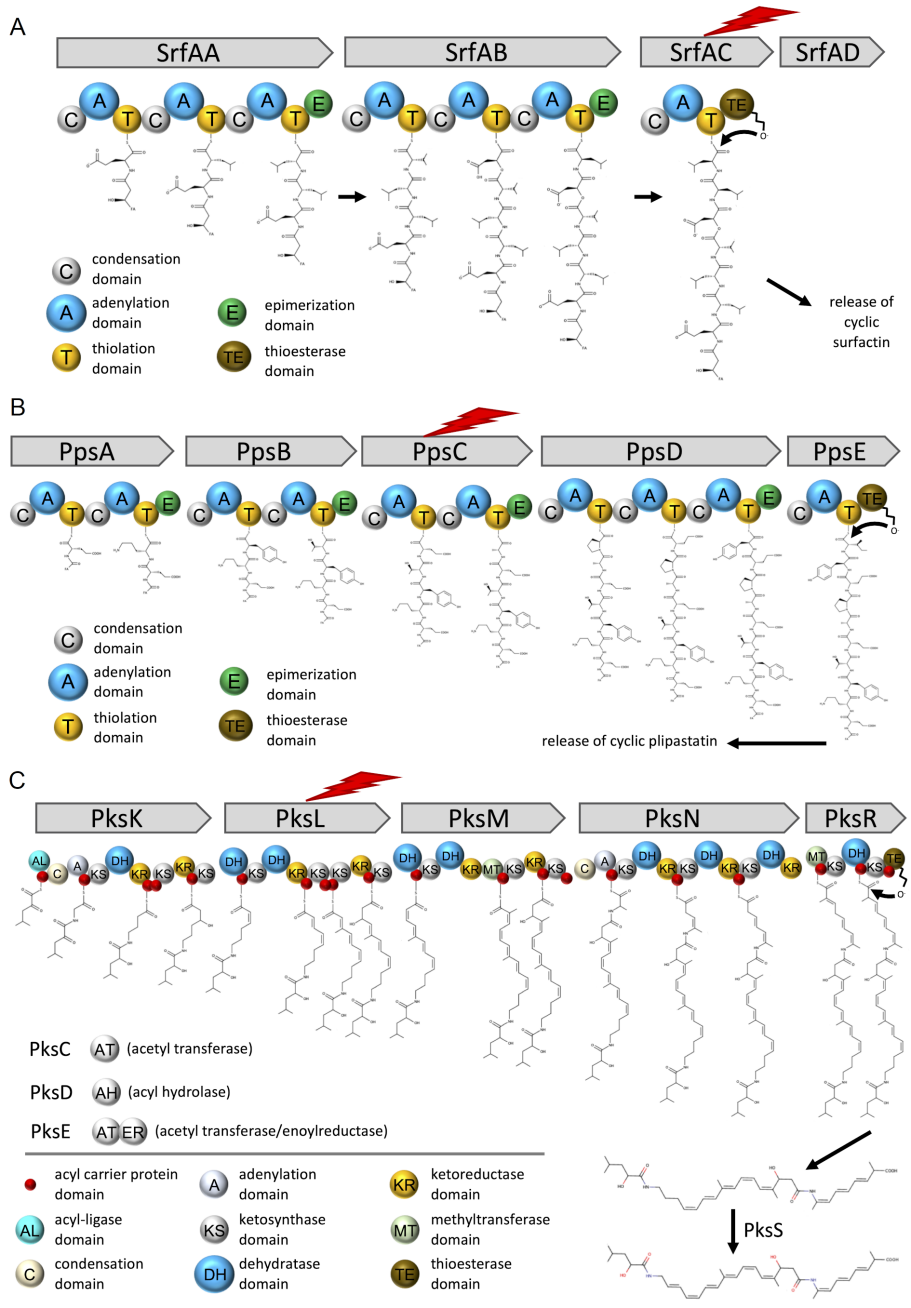
Overview on the biosynthetic pathways of surfactin (A), plipastatin (B), and bacillaene (C) produced by *B. subtilis*. The lightning bolt indicates the proteins for which the corresponding coding genes were deleted in the mutant strains.

*B. subtilis* is known to produce a wide range of different SMs that target a large number of various micro- and macroorganisms [35]. Our study demonstrates that the NRPs produced by the recently isolated environmental strain of *B. subtilis* P5_B1 did not strongly impact the overall soil-derived semisynthetic mock community but reduced the abundance of the genera *Lysinibacillus* and *Viridibacillus* (Figure 9). Moreover, it reveals that the strain *L. fusiformis* M5 was directly affected by the *B. subtilis* lipopeptide surfactin in a monitored growth experiment.

We studied the bacterial community compositions by sequencing the two variable regions V3 and V4 of the 16S rRNA gene. Noteworthy, some limitations of this technique are well known. In 2014, Poretsky et al. revealed that amplicon sequencing of the 16S rRNA gene indicates a lower sequence diversity and substantial differences in the relative abundances of specific genus-assigned taxa compared to metagenomics [55]. Moreover, 16S amplicon sequencing of single variable regions rarely allows sufficient discrimination below the family or genus level, and therefore intragenus differentiation and heterogeneity cannot be addressed [55]. Furthermore, the fundamental problem is that bacteria harbour various copy numbers of the 16S rRNA gene in the genomes, which biases quantification studies [56]. Alpha diversity analyses based on the Shannon estimation revealed that diversity was strongly reduced in in vitro cultivations. Furthermore, it was disclosed that the precultured soil suspension had the lowest diversity index because mainly the genera *Bacillus* and *Acinetobacter* were enriched, which can probably be traced back to different growth rates among the present species. A substantial shift in the community compositions was observed between in vivo and in vitro communities since the majority of the genera present in the in vitro communities was below the detection limit in the soil sample. However, during the 12 h precultivation of the soil suspension, bacteria were exposed to different nutrient availabilities, changed physical conditions, such as the temperature, a liquid environment, and the loss of the spatial soil structure. These conditions were most likely selecting for generalist bacteria capable of proliferating under the given conditions and independently from other bacteria. During the following 48 h cocultivation, depletion of the primary nutrient sources and metabolic cross-feeding further shaped the community assembly. In 2018, Goldford et al. revealed that the main sources of metabolic cross-feeding are secreted metabolic byproducts from the community members [57]. They further highlighted that bacterial communities stabilised after approximately eight to nine 48 h cocultivations. In our study, bacterial communities were only cocultivated once for 48 h, suggesting that the assembly of the bacterial communities has not yet reached a stable phase, which explains the differences between the precultures and cocultivated mock communities.

The Shannon index showed no differences among the established and differently treated mock communities, which primarily consisted of 13 genera. Even though *Bacillus* was the most abundant genus in the precultures, further incubation for 48 h resulted in a decreased relative abundance independently if the respective *B. subtilis* strains were seeded or the precultures were untreated. It shows that the initial dominance of *Bacillus* could not be maintained at prolonged incubation. The *B. subtilis* strains were added at a community assembly phase when *Bacillus* was the dominating genus, so that the general genera distribution was not expected to be influenced extensively. Nevertheless, after 48 h cocultivation, the final relative abundance of the *Bacillus* genus was not increased in the communities treated with *B. subtilis* when compared to the control. This observation highlights that the presence or absence of NRPs did not affect the competitiveness of *B. subtilis*. However, the 16S amplicon sequencing did not allow the detection of interactions and competitions within the *Bacillus* genus. The composition of this genus could vary among the differently treated communities. Nonetheless, the beta diversity analysis indicated a dissimilarity between the untreated and treated mock communities. Besides, two of the communities treated with the *sfp* mutant showed the highest similarity to the untreated communities, suggesting that the supplementation of the NRP-producing *B. subtilis* strains affected the communities. The vectors of *Acinetobacter* ASVs had a direction either to NRP-treated or NRP-untreated communities, indicating that the NRPs influenced species within the same genus differently.

In microbial communities, the amount of interactions and relations increases with the number of community members. The established semisynthetic mock communities in this study contained at least 13 genera with a relative abundance >0.19%. Therefore, it can be assumed that various interactions between them occurred. Nevertheless, we could observe statistically significant reductions of the two genera, *Lysinibacillus* and *Viridibacillus*, in communities supplemented with the NRP-producing *B. subtilis* wild type strain. In contrast, in communities supplemented with the NRP-deficient mutant *sfp*, *Lysinibacillus* was more frequent than in the wild type-treated communities. This observation indicates that NRPs have a great impact on suppressing *Lysinibacillus*. However, further factors are involved in the suppression since the *sfp* mutant maintained a reduction of *Lysinibacillus*, even though to a weaker extent. Moreover, no particular NRP could be allocated to the inhibition of the *Lysinibacillus* genus in these semisynthetic communities, but bacillaene displayed the highest impact on the suppression. An inhibition of *Viridibacillus* mediated by NRPs was also observable, but for this genus, bacillaene had the lowest impact. However, these results must be interpreted with caution and need further investigations since *Viridibacillus* was one of the lowest abundant genera in the mock communities, and abundance calculations are sensitive to the depth of sequencing. Besides the suppression of *Lysinibacillus* and *Viridibacillus*, *Stenotrophomonas* was uniquely suppressed in the communities supplemented with the *sfp* mutant but not when the WT strain was added. This observation might be evoked by inhibiting other species, which in turn facilitates a lower inhibition of *Stenotrophomonas*.

Previous studies revealed that the introduction of SM-producing bacteria to a bacterial community had no major impact on the entire composition. The tropodithietic acid-producing marine bacterium *Phaeobacter inhibens* did not strongly influence the microbiome diversity of the oyster *Ostrea edulis* but reduced the relative abundance of the orders Vibrionales and Mycoplasmatales [58]. Similar results were achieved when *B. velezensis* FZB42 was successfully applied as a biocontrol agent to lettuce in soil [59]. The authors could not see a substantial impact on the rhizosphere bacterial community by the supplemented biocontrol strain, whereas the sampling time and additional inoculation of the fungal plant pathogen influenced the community to a greater extent. Apart from soluble SM, volatile organic compounds (VOCs) are as well capable of impacting a microbial community. In 2020, Cosetta et al. demonstrated that VOCs of cheese rind-associated fungi have both growth-stimulating and -inhibiting properties on members of the rind microbiome [60]. The authors could reveal that the VOC-mediated shift of the bacterial community was caused due to growth promotion of *Vibrio* spp. These studies and the results from the semisynthetic mock community experiment of this study highlight that the overall impact of SMs on the targeted microbial communities is low, which suggests that they are no mass destruction compounds. However, in all communities, distinct genera or species were suppressed or promoted, emphasising the potential of SMs to shape microbial communities.

To investigate if *Lysinibacillus* is sensitive to any particular NRP of *B. subtilis*, we exposed the isolate *L. fusiformis* M5 to the spent media of the respective *B. subtilis* strains and monitored the growth. *L. fusiformis* M5 has been isolated from soil and demonstrated to impact the biofilm colony development of *B. subtilis* [54]. Interestingly, the modulation of the biofilm development was mediated by the primary metabolite hypoxanthine secreted by *L. fusiformis*. Of note, the impact of *B. subtilis* was not noticed on *L. fusiformis* in the mixed colony biofilm communities, possibly due to the use of the NRP-negative *B. subtilis* strain 168, which harbours a spontaneous frameshift mutation in the *sfp* gene [54]. Testing the impact of the natural isolate *B. subtilis* P5_B1 and the corresponding NRP mutant derivatives revealed that the spent media from both the NRP-deficient strain *sfp* and the surfactin-deficient strain *srfAC* had the lowest impact on the growth of *L. fusiformis*. In addition, the spent media of ∆*ppsC* and ∆*pksL* maintained the bioactivity at low concentrations, whereas the effect of WT was already strongly reduced at this level of the spent medium. This difference could occur, on the one hand, due to higher levels of surfactin in the two mutants compared to the wild type. On the other hand, the spent medium originated from cultures with an OD_600_ value of 3.0. Cultures with higher ODs were diluted before the harvesting, and WT cultures exhibited overall the highest ODs among the strains. Since the NRPs concentration is not proportional to the final OD due to, e.g., the occurrence of cell lysis, the spent media might be slightly differently diluted among the strains. Therefore, minor differences might be observable in the assays supplemented with highly diluted spent media. The observation that *L. fusiformis* displays a slightly higher cell density when the bacterial spent medium is supplemented might be due to the availability of additional nutrients. Nevertheless, the supernatant and pure compound supplementation demonstrated that surfactin is a direct suppressor of *L. fusiformis*. However, as the spent media of the *sfp* and *srfAC* strains still had a growth inhibition effect, it is plausible that next to surfactin, further NRPs and even other compounds might provoke a slight growth suppression of *Lysinibacillus*. When *L. fusiformis* was exposed to surfactin concentrations between 31.25 and 125 µg/mL, higher final cell densities were detectable compared to assays treated with higher levels of surfactin or in the control. Interestingly, in 2020, Arjes et al. demonstrated that surfactin enhances the availability of oxygen to *B. subtilis* by increasing the oxygen diffusivity [61], which might also positively affect the growth of *L. fusiformis*.

Experiments with differently treated semisynthetic mock communities have demonstrated that *Lysinibacillus* and *Viridibacillus* were affected by the addition of an NRPs-producing *B. subtilis* strain. *Lysinibacillus* was least affected in the mock communities supplemented with the *B. subtilis* ∆*pksL* strain incapable of producing bacillaene, suggesting that bacillaene is the most active compound against this genus. In contrast, the growth curve experiments showed that *L. fusiformis* M5 is most sensitive to surfactin. Importantly, our analysis does not reveal which *Lysinibacillus* species were present in the mock communities, and therefore their sensitivity might be different from the test species *L. fusiformis* used. Moreover, the spent medium was harvested from pure cultures of *B. subtilis* grown in an undiluted complex medium, which might have changed the production of NRPs due to the lacking impact of the community members and the level of nutrients. Thus, lower concentrations of the NRPs in the mock communities might affect *Lysinibacillus* differently compared to the monoculture growth experiments supplemented with spent media. Finally, *Lysinibacillus* can also be affected indirectly by *B. subtilis* NRPs in the mock communities. Bacillaene is described as a wide-spectrum antibiotic disrupting the protein synthesis in bacteria [34,52]. The observations suggest that it has the most substantial impact on specific members of the mock community, and consequently an indirect effect on *Lysinibacillus*. Nevertheless, the exact mechanisms at play remain to be deciphered.

Interestingly, the two genera *Lysinibacillus* and *Viridibacillus* of the mock communities are, besides *Paenibacillus*, the closest relatives of *B. subtilis*. The fact that suppression effects are only observable for these genera could presumably be caused by the higher overlap in the ecological niches, triggering competition for the same nutrients. Indeed, a higher phylogenetic and metabolic similarity between bacteria increases the probability of antagonism [62].

We could not quantify the concentrations of *B. subtilis* NRPs in the mock communities since the detection of low concentrations is still under development. However, a better understanding of their impact on the mock communities could be realised by further experiments investigating the effect of supplemented pure NRP compounds, e.g., surfactin and bacillaene. The impact of antibiotics on algae-associated bacterial communities was investigated by Geng et al. in 2016, who revealed a dose-depended influence of pure tropodithietic acid on the microbiome structure of *Nannochloropsis salina* [63]. Such pure NRP supplementations in various concentrations would allow exploring their effects on bacterial community assembly. Furthermore, in vivo experiments could reveal the impact of NRPs on microbial communities in complex natural systems, similar to the study from Chowdhury et al. from 2013 [59]. Noteworthy, our study focused only on NRPs, but additional SMs, such as bacteriocins, are predicted for *B. subtilis* P5_B1 as well [51]. Future investigations should investigate the impact of both bacteriocins and NRPs on microbial communities.

## Conclusion

In summary, this study demonstrates that nonribosomal peptides of *B. subtilis* P5_B1 have only a minor impact on the overall structure of soil-derived semisynthetic bacterial mock communities but suppress the genera *Lysinibacillus* and *Viridibacillus* significantly. Furthermore, it highlights the bioactivity of surfactin against *L. fusiformis* M5.

## Experimental

### Strains, media, and chemicals

All strains used in this study are listed in Table S1, Supporting Information File 1. For routine growth, bacterial cells were cultured in tryptic soy broth (TSB, CASO Broth, Sigma-Aldrich) containing 17 g⋅L^−1^ casein peptone, 3 g⋅L^−1^ soy peptone, 5 g⋅L^−1^ sodium chloride, 2.5 g⋅L^−1^ dipotassium hydrogen phosphate, and 2.5 g⋅L^−1^ glucose.

### Semisynthetic mock community assay

Semisynthetic soil communities were obtained from the soil of sampling site P5 (55.788800, 12.558300) [51,64]. 1 g soil was mixed in a 1:9 ratio with a 0.9% saline solution, vortexed on a rotary shaker for 15 min, and allowed to sediment for 2 min. Four independent communities were established by inoculating 10-times diluted TSB (0.1 × TSB) with 1% soil suspension taken from the middle part of the liquid phase, followed by incubation at 21–23 °C and 250 rpm for 12 h. Simultaneously, pregrown *B. subtilis* P5_B1 WT and the corresponding NRP mutant derivatives were inoculated in 0.1 × TSB and incubated in parallel using the same conditions. After 12 h precultivation, 3 mL aliquots of the soil suspension were transferred into six glass tubes. One tube was left untreated and functioned as control, whereas the remaining five were supplemented with respective *B. subtilis* strains by adding 10% of the final volume. The cultures were incubated at 21–23 °C and 250 rpm for 48 h. DNA was extracted from two replicates of the initial soil sample, the 12 h precultivated soil suspensions and the *B*. subtilis-treated or untreated mock communities cocultivated for 48 h.

### DNA extraction

Environmental- and semisynthetic-community genomic DNA was extracted from either 250 mg soil or 250 µL bacterial culture, respectively, by using the DNeasy PowerSoil Pro Kit (QIAGEN) and following the manufacturer’s instructions.

### Amplification of 16S rRNA hypervariable regions V3-V4

The V3-V4 region of the 16S rRNA gene was PCR-amplified from the extracted DNA samples using Fw_V3V4 (5’-CCTACGGGNGGCWGCAG-3’) and Rv_V3V4 (5’-GACTACHVGGGTATCTAATCC-3’) primers that were tagged with short barcodes with a length of eight nucleotides, listed in Table S2, Supporting Information File 1. The PCR reactions contained 10.6 μL DNase-free water, 12.5 μL TEMPase Hot Start 2x Master Mix, 0.8 μL of each primer (10 μM), and 0.3 μL of 50 ng/µL DNA template. The PCR was performed using the conditions of 95 °C for 15 min, followed by 30 cycles of 95 °C for 30 s, 62 °C for 30 s, 72 °C for 30 s, and finally, 72 °C for 5 min. All V3-V4 amplicons were purified using the NucleoSpin gel and PCR cleanup kit (Macherey-Nagel) and pooled in equimolar ratios. The amplicon pool was submitted to Novogene Europe Company Limited (United Kingdom) for high-throughput sequencing on an Illumina NovaSeq 6000 platform with 2 million reads (2 × 250 bp paired-end reads). Raw sequence data is available at NCBI: PRJNA658074.

### Sequencing data preprocessing

The multiplexed sequencing data was imported into the QIIME 2 pipeline (version 2020.6) [65–66]. The paired-end sequences were demultiplexed with the QIIME 2 plugin cutadapt [67]. The minimum overlap of partial matches between the read and the barcode sequence was set to 5 nucleotides to reduce random matches. The QIIME 2 implementation DADA2 was used to denoise and merge paired-end reads [68]. In total, 362,475 reads were assigned to the respective samples with an average of 12,083 reads per sample (range: 751 to 34,802; Table S3, Supporting Information File 1). The 16S rRNA reference sequences with a 99% identity criterion obtained from the SILVA database release 132 were trimmed to the V3-V4 region, bound by the primer pair used for amplification, and the product length was limited to 200–500 nucleotides [69]. The taxonomy was assigned to the sequences in the feature table generated by DADA2 by using the VSEARCH-based consensus taxonomy classifier [70]. A tree for phylogenetic diversity analyses was generated with FastTree 2 from the representative sequences [71–73].

### Relative species abundance and phylogenetic diversity analyses

QIIME 2 artefacts were imported into the R software (4.0.2) with the R package qiime2R, and further analyses were conducted in the R package phyloseq [74–76]. The taxonomy summaries were achieved by merging ASVs of the same genera and calculating their relative abundance in each sample. Differences in the presence of the most abundant genera in the control communities, in the communities supplemented with *B. subtilis* WT as well as in the communities supplemented with *B. subtilis sfp*, were investigated by calculating the abundance ratios of the different treated communities for each replicate. If species were not detected in some of the replicates, 0 values were replaced with the lowest detected value of the genus to avoid infinite values or 0 values in the ratio calculations. Rarefaction curves of the samples were calculated and visualised with the R package ranacapa [77]. Diversity analyses of the *B. subtilis*-treated and untreated samples were performed with ASV counts multiplied by factor 100,000 and transformed into integer proportions. The alpha diversity was estimated with the Shannon diversity index in the R package phyloseq [76]. The beta diversity was determined by dissimilarities among the samples with the Bray–Curtis distance and visualised in a nMDS with the R package vegan [78]. The correlation of individual ASVs on the overall bacterial community composition was calculated with the envfit function with 999 permutations from the R package vegan. The most correlating (*R*^2^ > 0.6) ASVs were added to the nMDS ordination plot. All graphical visualisations were realised with ggplot2 [79].

### Statistical analysis

The statistical significance was determined with the square roots of the tested values. The normality and equality of the variances were tested with the Shapiro–Wilk normality test and the Levene test, respectively. If one of the tests was rejected, the nonparametric Kruskal–Wallis rank sum test was performed instead. The statistical significance of pairs was determined with the Welch two-sample t-test, and the differences among groups >2 was determined with the one-way analysis of variance (ANOVA) test and the Tukey HSD test. The statistical significance was determined with an alpha level <0.05.

### Growth monitoring of *L. fusiformis* supplemented with *B. subtilis* spent media and pure surfactin

Spent media of *B. subtilis* strains were harvested from cultures grown in TSB medium at 37 °C and 250 rpm for 48 h immediately before the growth experiments. The cultures were adjusted to OD_600_ 3.0 and centrifuged for 4 min at 5,000*g*. Subsequently, the supernatants were passed through 0.22 µm filters and stored at 4 °C. The growth experiments were performed in 96-well microplates. The wells of the first column were filled with 30 µL 10 × TSB, 30 µL *L. fusiformis* culture adjusted to OD_600_ 0.1 in 1 × TSB, and 240 µL of the appropriate spent *B. subtilis* medium or water (untreated control). 100 µL *L. fusiformis* culture adjusted to OD_600_ 0.01 in 1 × TSB was added to the wells of the remaining columns. A 1.5-fold serial dilution of the spent media was performed column-by-column. A surfactin stock solution was prepared by dissolving 10 mg of surfactin (Sigma-Aldrich) in 1 mL methanol (MeOH). The wells of the first column were filled with 170 µL 1 × TSB, 20 µL *L. fusiformis* culture adjusted to OD_600_ 0.1 in 1 × TSB, and 10 µL surfactin, 10 µL MeOH (solvent control), or 10 µL 1 × TSB (untreated control). To the wells of the remaining columns, 100 µL *L. fusiformis* culture was added adjusted to OD_600_ 0.01 in 1 × TSB. A 2-fold serial dilution of surfactin or MeOH was performed column-by-column. In both assays, the growth of *L. fusiformis* was monitored in a microplate reader (BioTek Synergy HTX Multi-Mode Microplate Reader). The microplates were incubated at 30 °C with continuous shaking (548 cpm, 2 mm), and the OD_600_ was measured in 15 min intervals over 24 h. All graphical visualisations were prepared using ggplot2 [79].

## Supporting Information

File 1 Bacterial strains used in this study, 16S rRNA V3-V4 primer list, number of sequencing reads per sample, and supporting figures.
