# Supplementary material for: Secondary metabolites of Bacillus subtilis impact the assembly of soil-derived semisynthetic bacterial communities
Source: Beilstein J Org Chem. 2020 Dec 4;16:2983–98. doi: 10.3762/bjoc.16.248 (PMC7722629; doi:10.3762/bjoc.16.248)
Supplement: File 1 — Bacterial strains used in this study, 16S rRNA V3-V4 primer list, number of sequencing reads per sample, and supporting figures. [file Beilstein_J_Org_Chem-16-2983-s001.pdf]

BEILSTEIN JOURNAL OF ORGANIC CHEMISTRY

Supporting Information

for

Secondary metabolites of *Bacillus subtilis* impact the assembly of soil-derived semisynthetic bacterial communities

Heiko T. Kiesewalter, Carlos N. Lozano-Andrade, Mikael L. Strube and Ákos T. Kovács

Beilstein J. Org. Chem. **2020,** *16,* 2983–2998. doi:10.3762/bjoc.16.248

Bacterial strains used in this study, 16S rRNA V3-V4 primer list, number of sequencing reads per sample, and supporting figures

License and Terms: This is a supporting information file under the terms of the Creative Commons Attribution License (https://creativecommons.org/licenses/by/4.0). Please note that the reuse, redistribution and reproduction in particular requires that the author(s) and source are credited and that individual graphics may be subject to special legal provisions.
The license is subject to the *Beilstein Journal of Organic Chemistry* terms and conditions: (https://www.beilstein-journals.org/bjoc/terms)

Supporting tables

Table S1. Bacterial strains used in this study.

Strains	Characteristics	Reference
P5_B1	*B. subtilis* soil isolate from sampling site 55.788800, 12.558300	[1]
DTUB55	P5_B1 *sfp*::mls	[2]
DTUB79	P5_B1 *srfAC*::Tn*10* ($Spec^R$)	[1]
DTUB102	P5_B1 Δ*pksL* (Chl^R)	[2]
DTUB125	P5_B1 Δ*ppsC* (Tet^R)	[2]
M5	*Lysinibacillus fusiformis*	[3]

REFERENCES

1. Thérien, M.; Kiesewalter, H. T.; Auria, E.; Charron-Lamoureux, V.; Wibowo, M.; Maróti, G.; Kovács, Á. T.; Beauregard, P. B. *Biofilm* **2020**, *2*, 100021.

2. Kiesewalter, H. T.; Lozano-Andrade, C. N.; Wibowo, M.; Strube, M. L.; Maróti, G.; Snyder, D.; Jørgensen, T. S.; Larsen, T. O.; Cooper, V. S.; Weber, T.; Kovács, Á. T. *bioRxiv* **2020**, 2020.08.05.238063.

3. Gallegos-Monterrosa, R.; Kankel, S.; Götze, S.; Barnett, R.; Stallforth, P.; Kovács, Á. T. *J. Bacteriol.* **2017**, *199*, e00204-17.

Table S2. 16S rRNA V3-V4 primer used in this study

**Name**	**Barcode**	**Primer**
V3V4_forw_1	TTTTAATC	CCTACGGGNGGCWGCAG
V3V4_rev_1	TTTTAATC	GACTACHVGGGTATCTAATCC
V3V4_forw_2	ATAATTAG	CCTACGGGNGGCWGCAG
V3V4_rev_2	ATAATTAG	GACTACHVGGGTATCTAATCC
V3V4_forw_3	ACCAAATT	CCTACGGGNGGCWGCAG
V3V4_rev_3	ACCAAATT	GACTACHVGGGTATCTAATCC
V3V4_forw_4	CTTATCAA	CCTACGGGNGGCWGCAG
V3V4_rev_4	CTTATCAA	GACTACHVGGGTATCTAATCC
V3V4_forw_5	TGATCATT	CCTACGGGNGGCWGCAG
V3V4_rev_5	TGATCATT	GACTACHVGGGTATCTAATCC
V3V4_forw_6	AGAATCTA	CCTACGGGNGGCWGCAG
V3V4_rev_6	AGAATCTA	GACTACHVGGGTATCTAATCC
V3V4_forw_7	TCAAGAAA	CCTACGGGNGGCWGCAG
V3V4_rev_7	TCAAGAAA	GACTACHVGGGTATCTAATCC
V3V4_forw_8	ATCGAAAT	CCTACGGGNGGCWGCAG
V3V4_rev_8	ATCGAAAT	GACTACHVGGGTATCTAATCC
V3V4_forw_9	ACATTTAC	CCTACGGGNGGCWGCAG
V3V4_rev_9	ACATTTAC	GACTACHVGGGTATCTAATCC
V3V4_forw_10	TAGAAAAC	CCTACGGGNGGCWGCAG
V3V4_rev_10	TAGAAAAC	GACTACHVGGGTATCTAATCC
V3V4_forw_11	TTATCACC	CCTACGGGNGGCWGCAG
V3V4_rev_11	TTATCACC	GACTACHVGGGTATCTAATCC
V3V4_forw_12	AATAGGGT	CCTACGGGNGGCWGCAG
V3V4_rev_12	AATAGGGT	GACTACHVGGGTATCTAATCC
V3V4_forw_13	ATTGCTGA	CCTACGGGNGGCWGCAG
V3V4_rev_13	ATTGCTGA	GACTACHVGGGTATCTAATCC
V3V4_forw_14	TGAGTTCT	CCTACGGGNGGCWGCAG
V3V4_rev_14	TGAGTTCT	GACTACHVGGGTATCTAATCC
V3V4_forw_15	GGCTATTT	CCTACGGGNGGCWGCAG
V3V4_rev_15	GGCTATTT	GACTACHVGGGTATCTAATCC
V3V4_forw_16	CAAGAGAT	CCTACGGGNGGCWGCAG
V3V4_rev_16	CAAGAGAT	GACTACHVGGGTATCTAATCC
V3V4_forw_17	GGAATACA	CCTACGGGNGGCWGCAG
V3V4_rev_17	GGAATACA	GACTACHVGGGTATCTAATCC
V3V4_forw_18	AAGGCAAT	CCTACGGGNGGCWGCAG
V3V4_rev_18	AAGGCAAT	GACTACHVGGGTATCTAATCC
V3V4_forw_19	ACAAAACG	CCTACGGGNGGCWGCAG
V3V4_rev_19	ACAAAACG	GACTACHVGGGTATCTAATCC
V3V4_forw_21	TTGAGTGA	CCTACGGGNGGCWGCAG
V3V4_rev_21	TTGAGTGA	GACTACHVGGGTATCTAATCC
V3V4_forw_22	GCTTCTGA	CCTACGGGNGGCWGCAG
V3V4_rev_22	GCTTCTGA	GACTACHVGGGTATCTAATCC
V3V4_forw_23	GGCAAGAT	CCTACGGGNGGCWGCAG
V3V4_rev_23	GGCAAGAT	GACTACHVGGGTATCTAATCC

V3V4_forw_24	GTGCTTTC	CCTACGGGNGGCWGCAG
V3V4_rev_24	GTGCTTTC	GACTACHVGGGTATCTAATCC
V3V4_forw_25	ACACACTG	CCTACGGGNGGCWGCAG
V3V4_rev_25	ACACACTG	GACTACHVGGGTATCTAATCC
V3V4_forw_26	CGATTCTG	CCTACGGGNGGCWGCAG
V3V4_rev_26	CGATTCTG	GACTACHVGGGTATCTAATCC
V3V4_forw_27	GCAGAGTT	CCTACGGGNGGCWGCAG
V3V4_rev_27	GCAGAGTT	GACTACHVGGGTATCTAATCC
V3V4_forw_28	CGTCCTAT	CCTACGGGNGGCWGCAG
V3V4_rev_28	CGTCCTAT	GACTACHVGGGTATCTAATCC
V3V4_forw_30	GCTTGGTT	CCTACGGGNGGCWGCAG
V3V4_rev_30	GCTTGGTT	GACTACHVGGGTATCTAATCC
V3V4_forw_31	ACAGGCTT	CCTACGGGNGGCWGCAG
V3V4_rev_31	ACAGGCTT	GACTACHVGGGTATCTAATCC
V3V4_forw_33	TGACGCTT	CCTACGGGNGGCWGCAG
V3V4_rev_33	TGACGCTT	GACTACHVGGGTATCTAATCC

Table S3. Number of reads per sample.

Sample	Number of reads
Soil 1	751
Soil 2	12,660
Pre 1	20,440
Pre 2	17,787
Pre 3	15,471
Pre 4	34,802
Control 1	4,649
Control 2	20,846
Control 3	16,229
Control 4	16,556
WT 1	12,436
WT 2	18,554
WT 3	18,182
WT 4	11,964
sfp 1	13,354
sfp 2	16,737
sfp 3	12,472
sfp 4	21,421
srfAC 1	7,047
srfAC 2	1,184
srfAC 3	3,563
srfAC 4	7,663
Δ*pksL* 1	11,017
Δ*pksL* 2	2,115
Δ*pksL* 3	7,063
Δ*pksL* 4	5,187
Δ*ppsC* 1	9,443
Δ*ppsC* 2	10,823
Δ*ppsC* 3	9,947
Δ*ppsC* 4	2,112

Supporting figures

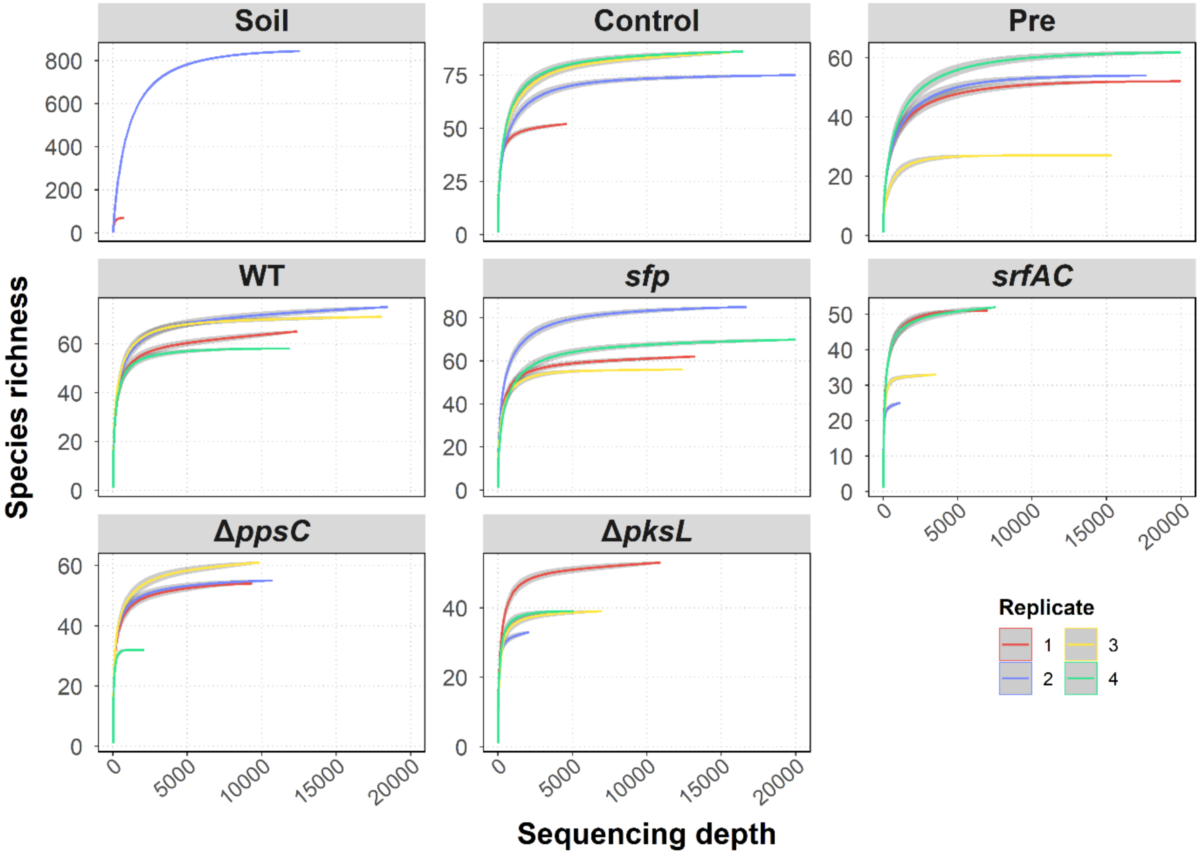

Figure S1. Rarefaction curves for each assay and replicate. Species richness is visualised for sampling depths from 0 to 20,000.

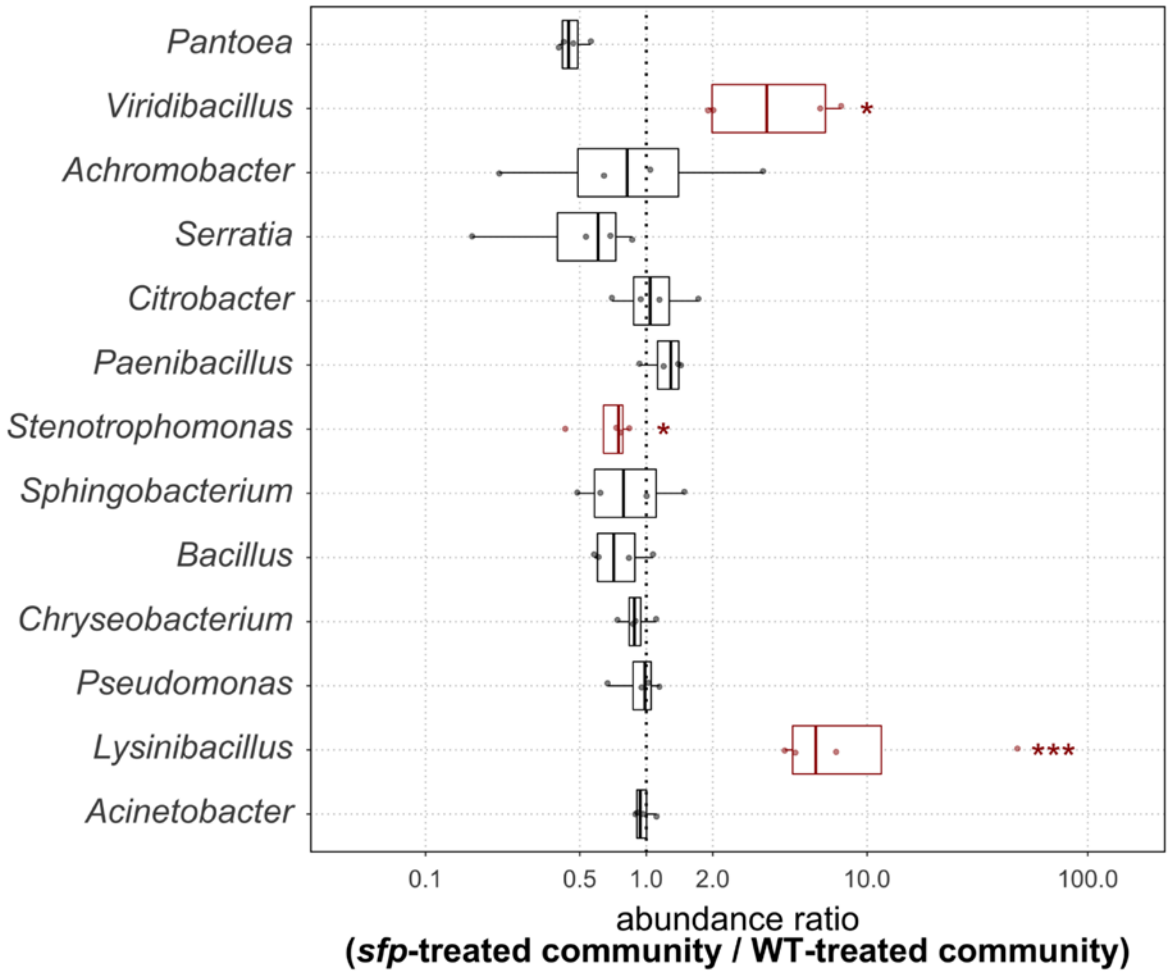

Figure S2. Abundance ratios for each genus and replicate (points) in the *sfp*-treated community compared to the WT-treated community. Statistical significance is defined as $P \leq 0.05$ (*) and $P \leq 0.001$ (***).

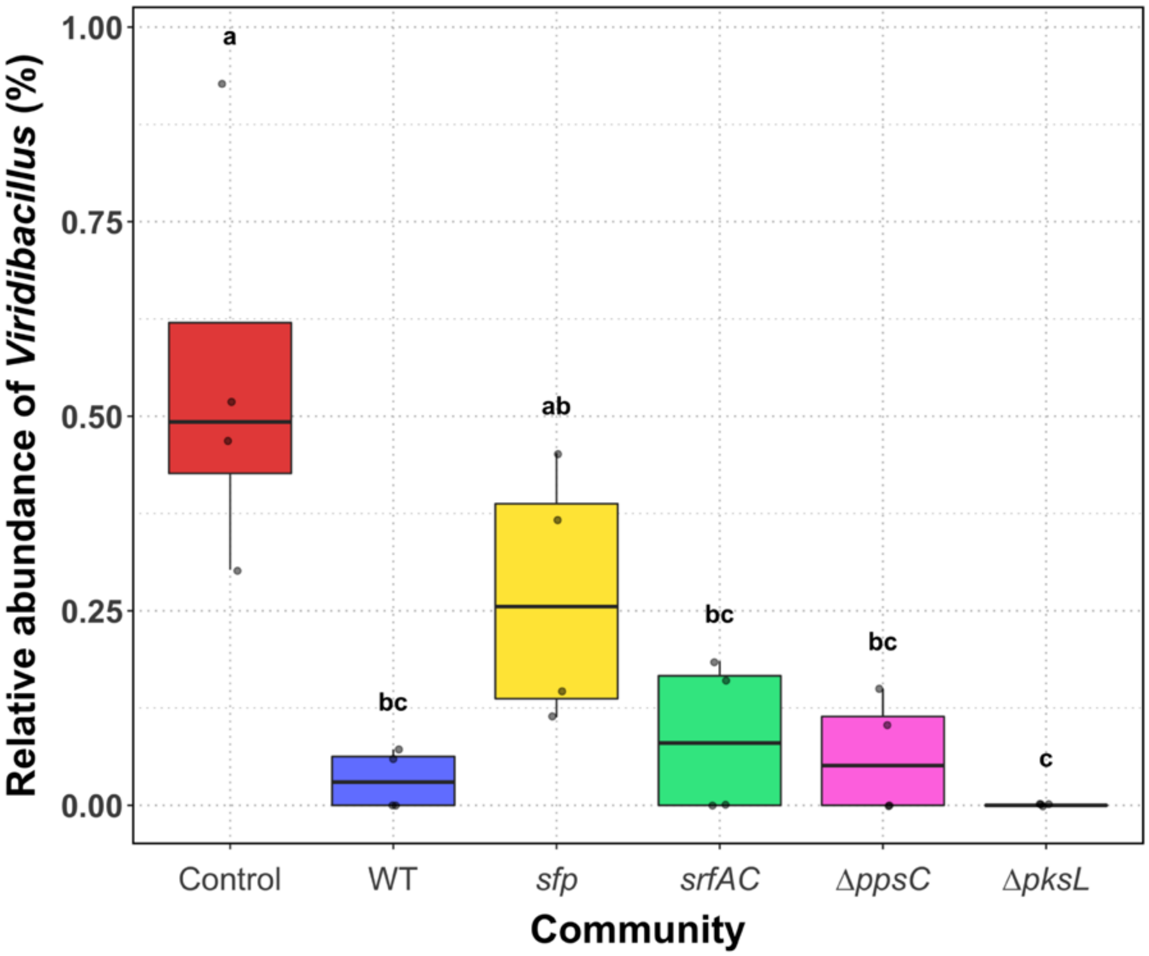

Figure S3. Abundance of *Viridibacillus* in the untreated mock communities (Control) and treated mock communities with either *B. subtilis* wild type (WT), NRPs deficient strain (*sfp*), surfactin mutant (*srfAC*), plipastatin mutant (Δ*ppsC*) or bacillaene mutant (Δ*pksL*) and co-cultivated for 48 h. Points represent the abundance in each replicate. Treatments with varying letters are significantly different ($P < 0.05$).

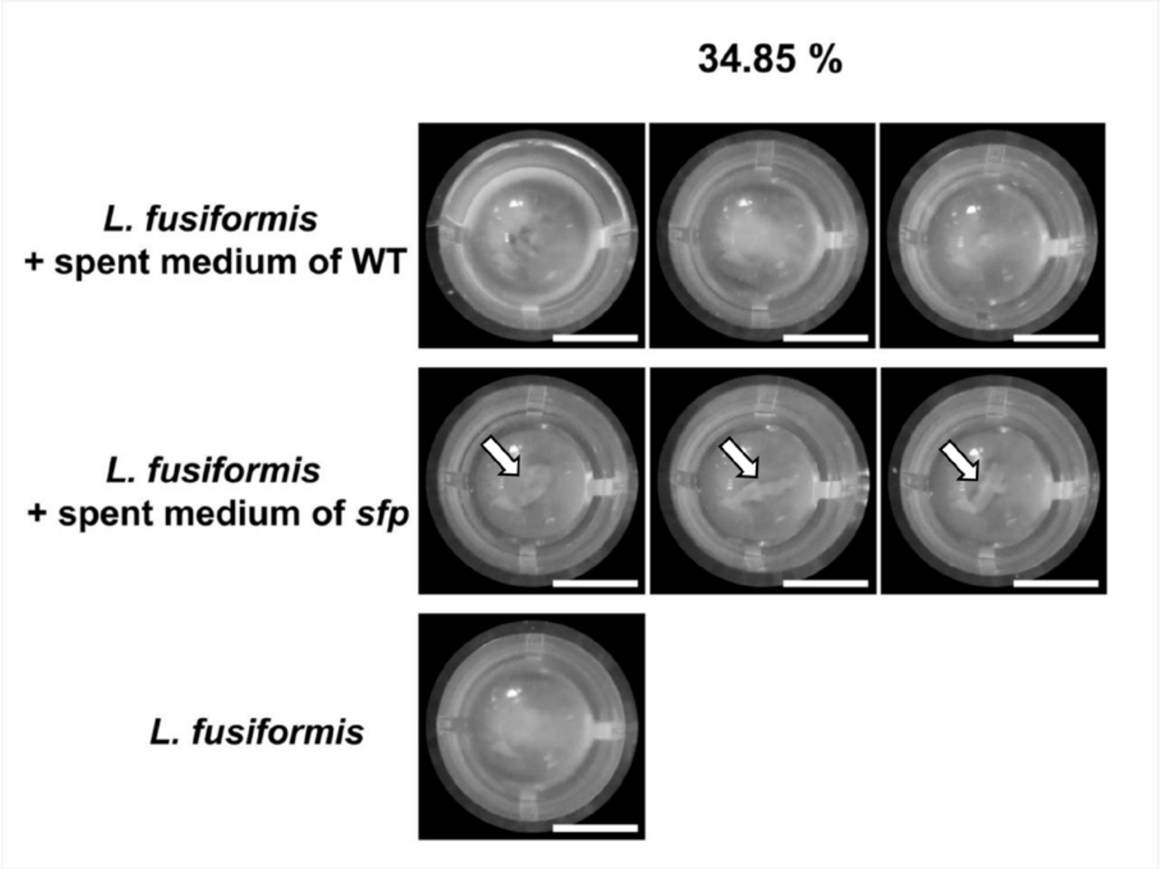

Figure S4. Different levels of aggregation of *L. fusiformis* M5 after exposure to spent medium of *B. subtilis* WT and *sfp* or without treatment. Solid aggregates in assays supplemented with spent medium of *sfp* are marked with arrows. Scale bars indicate 3 mm.

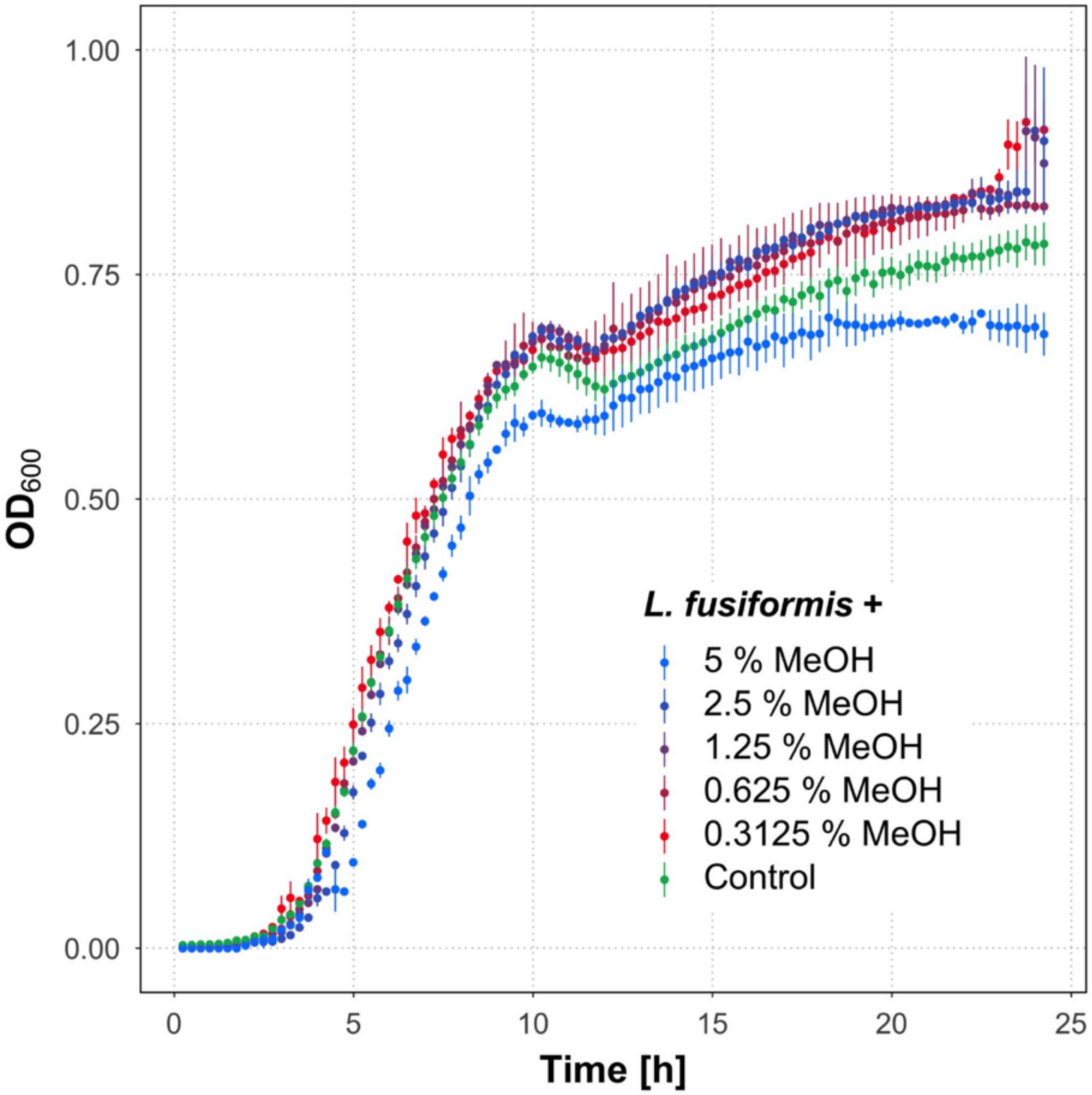

Figure S5. Growth curves of *L. fusiformis* M5 exposed to different concentrations of the solvent MeOH and without treatment (control). Error bars represent the standard error. N = 6 (control assay), N = 2 (MeOH-treated assays)
